# Feasibility of Use of a Mobile Application for Nutrition Assessment Pertinent to Age-Related Macular Degeneration (MANAGER2)

**DOI:** 10.1167/tvst.6.1.4

**Published:** 2017-01-20

**Authors:** Zaria Christine Ali, Richard Silvioli, Azita Rajai, Tariq Mehmood Aslam

**Affiliations:** 1 Manchester Royal Eye Hospital, CMFT, Manchester Academic Health Sciences Centre, Manchester, UK; 2 RS Nutrition and Dietetics Ltd.7 Bickley Close, Hough, UK; 3 Institute of Population Health, Faculty of Medical and Human Sciences, University of Manchester, Manchester, UK; 4 Research & Innovation, Central Manchester University Hospitals NHS Foundation Trust, Manchester Academic Health Sciences Centre, Manchester, UK; 5 Division of Pharmacy and Optometry, School of Health Sciences, University of Manchester, Manchester, UK; 6 University of Manchester, Manchester, UK; 7 Heriot Watt University, Edinburgh Campus, Edinburgh, UK

**Keywords:** mobile application, nutrition, monitoring, AMD

## Abstract

**Purpose:**

This is a feasibility study assessing use of a mobile phone application (app.) to measure nutrient intake relevant to age-related macular degeneration (AMD).

**Methods:**

Inclusion criteria were age over 40 and ownership of a smartphone. Participants included healthy volunteers and those with ophthalmic conditions. They were asked to record daily food intake for a minimum of 3 days in a paper food diary and the app. A dietician analyzed the food diaries, and an independent researcher analyzed data from the app. Average daily intake of nutrients relevant to AMD (docosahexaenoic acid [DHA], eicosapentaenoic acid [EPA], vitamins E and C, copper, zinc, and lutein + zeaxanthin) were calculated for both and then compared.

**Results:**

A total of 54 participants completed the app. and food diary. Male-to-female ratio was 7:20. Median (interquartile range [IQR]) age was 57 years (45.3–68.7 years). More than 90% of all values were within the limits of agreement for all micronutrients. Bland Altman agreement plots demonstrated clinically acceptable agreement between the two systems of analysis.

**Conclusions:**

This study has demonstrated that the app. is a feasible alternative to the food diary for assessing nutrient intake relevant to AMD. Further studies are suggested to assess long-term adherence and effect of the app. on nutrient intake in AMD patients.

**Translational Relevance:**

After smoking, nutritional modification is the key modifiable factor to reduce incidence of AMD. Use of the app. could be an efficient, easy way to monitor and improve dietary intake of required nutrients pertinent to AMD.

## Introduction

Age-related macular degeneration (AMD) is the most common cause of visual impairment and blindness in industrialized nations^1^ and with an ageing population its prevalence is predicted to rise further. The development of advanced neovascular choroidal changes accounts for more than 80% of severe visual disability caused by AMD.^2^ Landmark studies have revolutionized care by demonstrating that intravitreal anti-vascular endothelial growth factor (anti-VEGF) can lead to far better visual function than has ever previously been possible,^3,4^ but the condition still involves a high clinical and socioeconomic burden with real-world outcomes currently far inferior to those of the landmark studies.^5^

Therefore, all options to slow down the rate of development of macular degeneration in patients at risk are still of utmost importance. After smoking, nutritional modification is the key modifiable factor to reduce incidence of AMD and no other preventative treatments currently are generally available.^6–8^ Multiple observational and interventional studies^7–10^ have shown higher levels of intake of the omega three fatty acids docosahexaenoic acid (DHA) and eicosapentaenoic acid (EPA), vitamins C and E, and also nutrients copper, zinc, and lutein and zeaxanthin to be associated with lower rates of macular degeneration. A good intake of these key micronutrients is often advised by ophthalmologists for patients at risk of AMD development and those who have AMD to prevent progression.^11^

Although dietary advice may be given, monitoring or measuring precise intake does not appear to be common practice and this is likely due to the challenges of achieving this goal. Common tools used by health professionals to assess diet for patients with chronic conditions include food diaries, 24-hour recall interviews, and questionnaires, but these methods have several limitations, such as being overly time-consuming, and reliant on patient memory and on the availability of dieticians to analyze data.^12^

Mobile phone software applications (“apps.”) may potentially provide some help in this area. Applications related to health have become increasingly popular and it has been suggested that up to 500 million people currently are using health related apps.^13^ In particular, health professionals have used apps. to monitor and encourage better lifestyle and dietary choices for conditions, such as diabetes and renal disease.^13–16^ The use of these apps. also has demonstrated increased adherence for dietary monitoring.^14,17^

The aim of this study was to assess the feasibility of using a mobile phone app. to measure nutrient intake relevant to AMD and to compare results of the nutrient levels to those from a gold standard method of dietary intake recorded by an established paper-based food diary.

## Materials and Methods

A prospective observational cohort study was done. Prospective approval for the study was gained from REC East of England, Cambridge South and informed consent was gained from all participants. Local registration number in Manchester is R03980.

A prototype mobile phone app. has been developed to provide an efficient and user friendly tool to measure a patient's nutrient intake relevant to AMD. It contains functions to allow easy input of a daily electronic food record and calculate weekly nutritional reports specifically pertinent to the prevention and progression of macular degeneration. The electronic food record is split into four categories for each day: breakfast, lunch, dinner, and snacks. Patients input all the foods they eat in a given day using a search function to help them find food items or meals from an extensive on-line database. If a food item is not within the database they can enter it as a new food, or forward a copy of the food label or recipe to the dietician who will add the food or meal to the database. Users also are able to save foods into a “favorites” section, allowing them to easily access frequently consumed foods. The app. calculates intake of key nutrients by accessing an extensive nutrient database. The app. was made available to download for Apple and android operating systems with access limited to selected users.

Participants were recruited to the study from within and outside the hospital service with simple inclusion criteria that participants must be aged 40 or over and that they must own a smartphone (android or Apple). The entry criteria were kept broad to reflect the range of subjects who might be interested in using this app.; for example, those with a family history, those with an established genetic risk and those with minor macular abnormalities as well as those with established macular degeneration.

Participants were asked to download the app. and record their daily food intake for a minimum of two weekdays and one weekend day in the smartphone app. They also were required to record their food intake in the same period using an established paper food diary. Each participant was given a unique username, password, and identification number.

**Figure 1:**
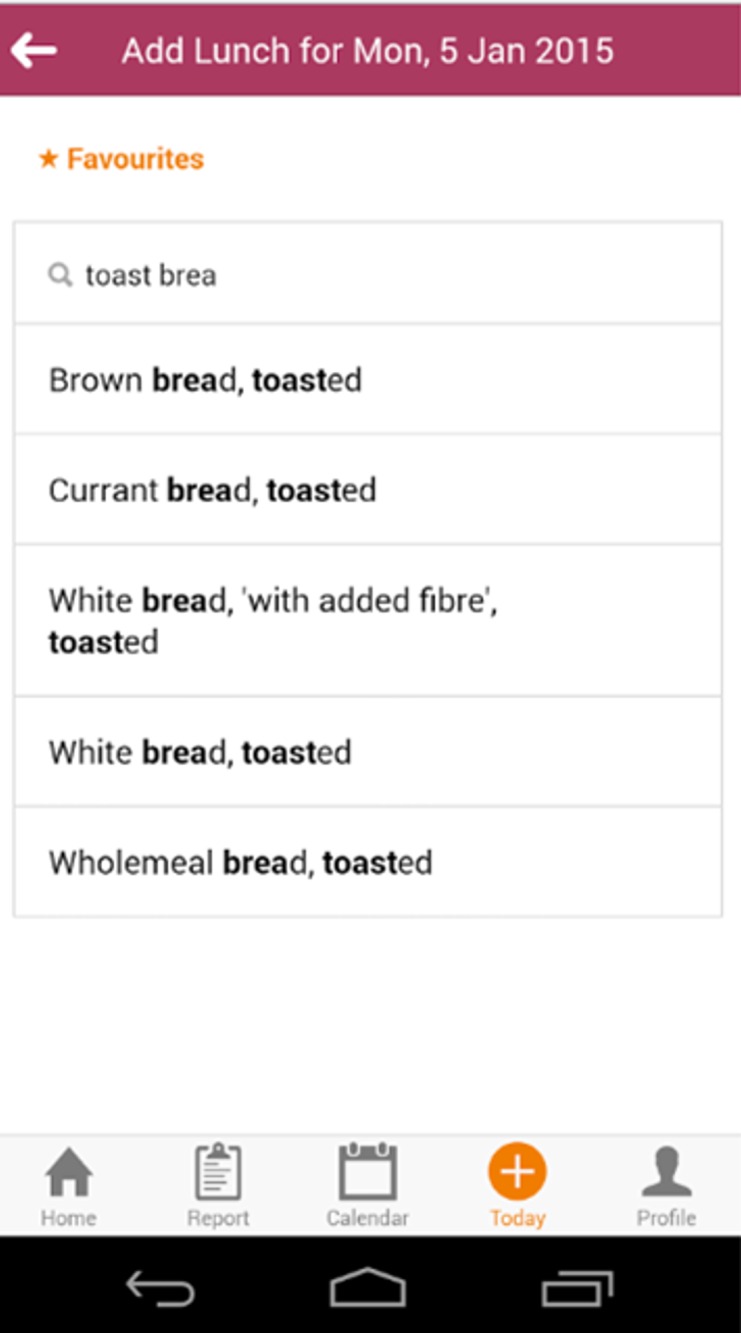
Example of app. interface. When a food is typed into the search bar suggestions from the app. database are loaded.

**Figure 2:**
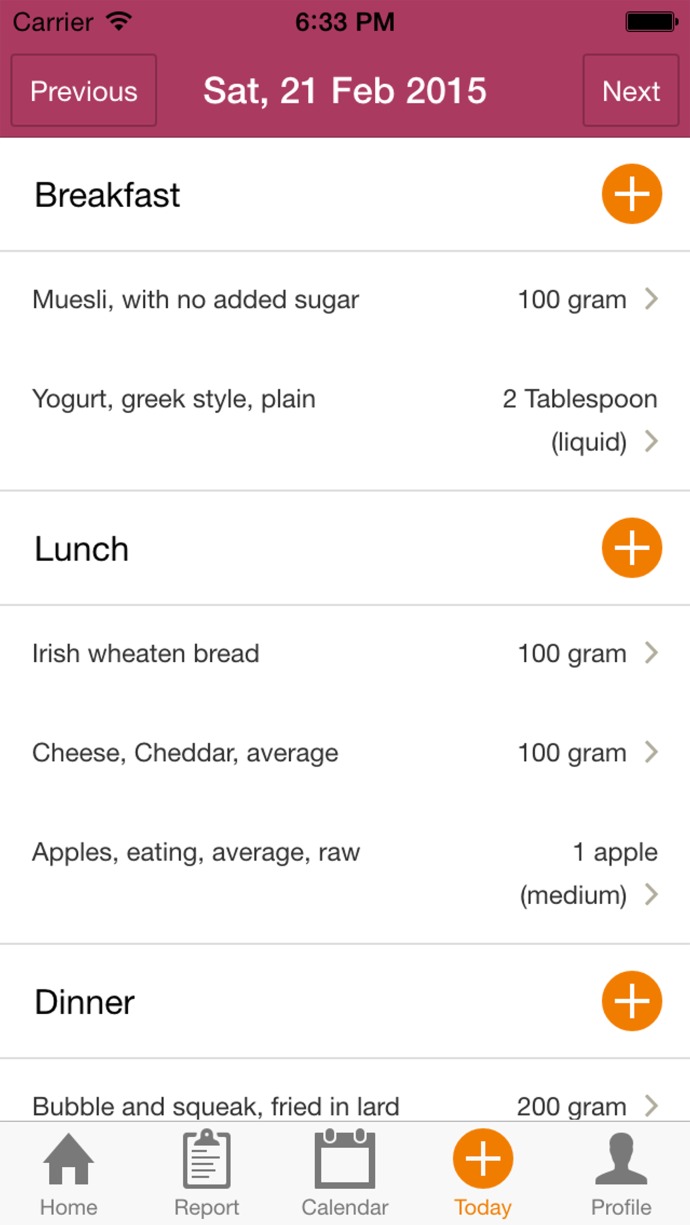
Example of app. interface. Participants must enter the different constituents that make up their meals.

Examples of the app. interface are shown in Figures 1 and 2. Information inputted to the app. was automatically uploaded to a central database which then could be accessed by a researcher independent of the dietician. Full patient confidentiality was maintained by each participant being given a unique identifier and uploaded information labelled only by this identifier.

Standardized paper food diaries were provided to each of the patients as used by Bartlett and Eperjesi,^18^ who have conducted a cross-sectional dietary analysis in normal and AMD patients. The diaries were labelled with the participants' identification number. A section at the beginning of the food diary illustrated examples of how their daily intake should be recorded with another section showing suggested portion sizes they could quote should they struggle to estimate weights.

Full instructions on how to fill in the paper food diary and app. were given and a contact number for the research coordinator was given for participants who required additional help. At the end of the study food diaries were returned to the dietician who discussed details of diet with the patient if necessary to clarify exact intake of food. Food diaries then were analyzed by the dietician who calculated the average daily intake of the omega 3 fatty acids DHA and EPA, vitamins E and C, copper, zinc, and lutein + zeaxanthin. An independent researcher recorded the average daily intakes of the same nutrients over the same dates, as calculated by the app. The corresponding values from the standard food diary and computer app. were compared for each of the nutrients.

**Figure 3:**
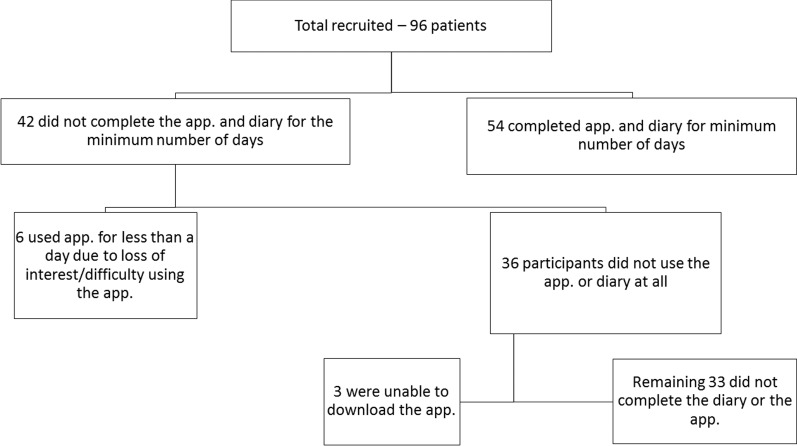
Breakdown of included and excluded patients.

We recruited a total of 96 patients: 36 did not use the app. at all, while a further 6 used it for less than a day (Fig. 3). These 42 patients were not included in analyses. For a short period of time the app. was unavailable to download due to technical difficulties, which led to 3 of the patients withdrawing. This problem was mitigated in other recruits by ensuring the app. was downloaded on the day of consent.

Bland-Altman plots with 95% agreement levels were used to measure the agreement between values found from the app. and paper diary for each nutrient. Statistical software “stats direct” was used. The limits of agreement were taken to be 2 standard deviations (SDs) from the mean difference. In addition, verbal feedback was obtained regarding use or lack of use of the app. and food diary.

## Results

A total of 54 participants completed the app. and the paper food diary for the minimum number of days. Male-to-female ratio was 7:20. Median (interquartile range [IQR]) age was 57 years (45.3–68.7 years). Mean (SD) number of days dietary intake was recorded at 4 (6) days. Of 13 participants with known ophthalmic conditions, 7 had AMD, 3 had diabetic retinopathy, and 3 had retinal vein occlusions. The remaining 41 did not have any formal diagnosis of an ophthalmic condition. Comparison of demographics of included patients and those who withdrew or were excluded are shown in Table 1.

**Table 1:**
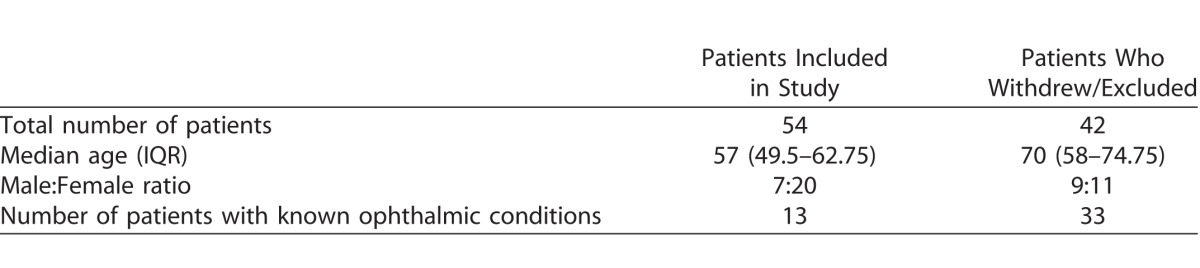
Differences in Demographics between Included and Excluded Patients

Just over a third of the 42 patients who withdrew (16) were younger than 60 years and working full time. Completing the food diary and the app. was cited as being too time-consuming. Loss of interest and the requirement for high data input also were common reasons for withdrawal. Other complaints and negative comments included difficulty using the search function, an “overly complicated” food database, limited number of items that can be added to the favorite section, and occasional “freezing” of the app. due to poor internet signal.

Despite difficulties with the app., there was a predominantly positive response; users felt the set portions sizes and favorite sections were useful and they also reported they were better able to understand and monitor their diet.

Bland–Altman agreement plots (supplementary Figs. S1–7) and summaries in Table 2 suggests clinically acceptable agreement between the two systems of analysis for all micronutrients.

**Table 2:**
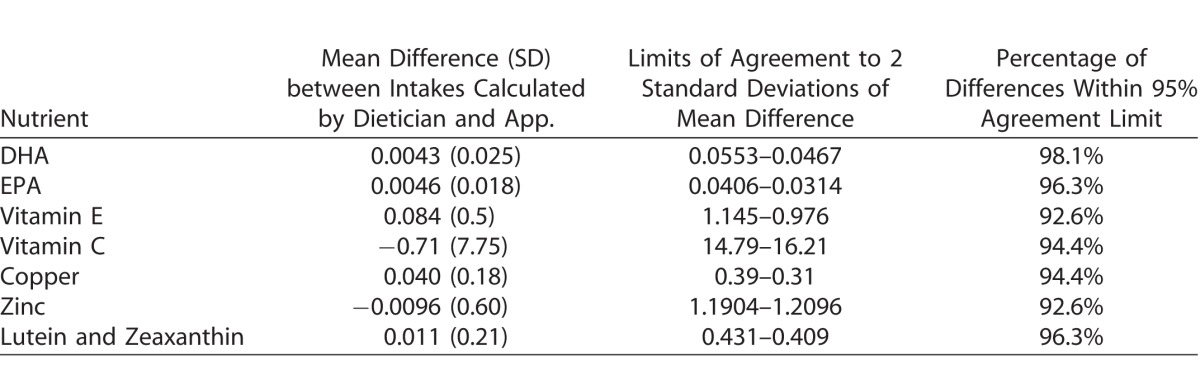
Summary of Comparisons of Nutrient Intakes by App. and Dietician

Outliers were checked and confirmed to be due to inevitable differences in the way food was recorded in the diary and app. which is explored further in the discussion.

## Discussion

Results demonstrated that the agreement between the app. calculations of nutrient intake and those derived from the diary were clinically acceptable. When accounting for outliers outside 95% agreement levels there were four common reasons for discrepancies. When using the app. patients were more specific with the type of food they ate, were more likely to enter a weight for the food, logged their snacks and drinks more reliably, and also were more likely to log constituents ingredients of a meal, such as dressings and type of oil used.

Although limits of agreement for vitamin C appear quite wide, the clinically acceptable levels for general vitamin C intake are quite large with the lowest recommended dietary allowance being 75 mg in females and 90 mg in males, and tolerable upper intake levels being up to 2000 mg for both sexes.^19^

Overall, it appeared that those patients who did complete the app. were able to produce very accurate results of nutrient intake through its software. This suggests that the precise user interfaces, coding, and nutrient databases that the app. was founded upon were correct. Feedback we received indicated that issues with app. use could be addressed comprehensively by improving the search function, simplifying the food database, and increasing number of foods that can be stored as favorites.

Improving in these areas could lead to a system of measurement using electronic apps. that matches the accuracy and usability of standard paper-based dietary monitoring, but with several key advantages. Firstly, it is accessible via a smartphone, which is more portable than a food diary, and allows patients to record their dietary intake easily throughout the day. As well as providing convenience, this avoids problems with recall. Previous studies have shown that adherence to self-monitoring is greater when using mobile apps. in comparison with recording diet using paper and pen.^14^

Furthermore, several qualitative studies looking at various health apps. demonstrated the positive attitude of patients using apps. This included feeling more motivated and also feeling the app. was important to them and benefited them.^20–22^ In addition, the app. automatically calculates the nutrient levels allowing health professionals to obtain data immediately. In contrast, each food diary required hours of work by a dietician to calculate relevant levels of nutrients. Finally, the potential to deliver personalized feedback is an important future role of the app. and such feedback has been reported as one of the most effective strategies to encourage change.^15,23,24^ In addition to its current function, the app. could give advice as to which foods to consume to address any dietary insufficiencies. Having a professional reference advising which foods to eat has been cited as an important app. feature in a study assessing the use of an app. to increase iron intake in pregnant women.^25^ Although apps. often can encourage and motivate patients they cannot completely replace counselling and support by a health professional.^23^ Thus, feedback from the patient's ophthalmologist will remain an important part of the process. In general, the use of the MANAGER app. could provide a practical, cheap and manageable means of accurately recording nutrient intake relevant to AMD and offers the potential benefits of personalized feedback.

There are several limitations to this study and success of the app. in patients who did complete the study must be balanced against high dropout rates. It can be seen that those who dropped out often were older patients. This may be due to lack of interest in the app. or struggling to understand the app. interface. However, it should be noted that the majority of these patients (33 of 42) did not use the paper diary either. This suggests they did not participate either due to lack of motivation, time constraints, or difficulties with their vision preventing them from using either the diary or app. Once issues with the app. interface are addressed as previously discussed, these patients may be more comfortable using the app. In terms of future use of the app., it is most likely to be of use for patients who are at very early stages or only at risk of AMD, who often would have good reading vision. Patients at later stages of AMD, with poor reading ability, might already be directed towards oral supplements rather than dietary change, reducing the need for this app. However, it would be useful for future versions of the app. to facilitate its use for any patients with reduced vision, for example by having options for larger text sizes. After these improvements are implemented further studies to assess app. use in patients who specifically wish to guard against AMD is warranted.

Long-term adherence also must be assessed. Although studies have shown that app. use has led to greater adherence to dietary monitoring,^14,17^ other studies have reported that app. use tends to reduce over time.^16^ Indeed, we found there was difficulty in encouraging patients to maintain use of the app. and diary for 3 days. This may have been expected from previous reported studies with high dropout rates; a cross-sectional survey in the United States looking at the use of health apps. reported more than half of participants stopped using the apps. due to loss of interest and the requirement for high data input.^26^

Although the results of this study showed that the app. is comparable to the food diary neither monitoring technique addresses issues, such as selective underreporting of foods and reporting bias.^12,27^ Correlation of nutrient intake on the app. with serum levels of patients may help assess the accuracy of their self-monitoring.

Finally, further investigation into the effect of the app. to encourage dietary change and increase micronutrient intake is warranted as a key ultimate objective of this technology.

## Conclusion

Before this study there was limited evidence regarding mobile app. use in the mid-elderly age group.^16^ To our knowledge, there were no studies assessing an app. measuring micronutrients relevant to AMD despite a clear potential clinical role. This study has revealed many challenges in design and further developments are required. However, it has demonstrated that the mobile app. is a feasible, practical, accurate, and rapid potential alternative to the food diary for assessing nutrient intake relevant to prevention and progression of AMD.

## Supplementary Material

Supplement 1

Supplement 2

Supplement 3

Supplement 4

Supplement 5

Supplement 6

Supplement 7
