# Supplementary figures and images for: Feasibility of Use of a Mobile Application for Nutrition Assessment Pertinent to Age-Related Macular Degeneration (MANAGER2)
Source: Transl Vis Sci Technol. 2017 Jan 20;6(1):4. doi: 10.1167/tvst.6.1.4 (PMC5270627; doi:10.1167/tvst.6.1.4)

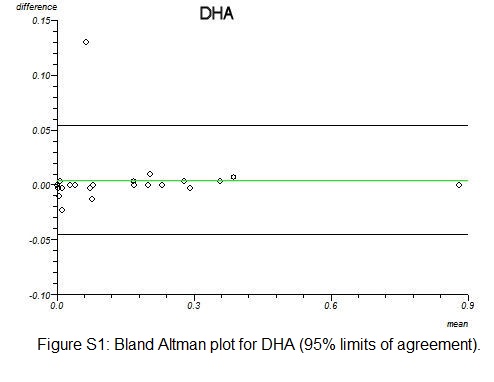

Supplement: Supplement 1 [file TVST-16-0395-s01.jpg]

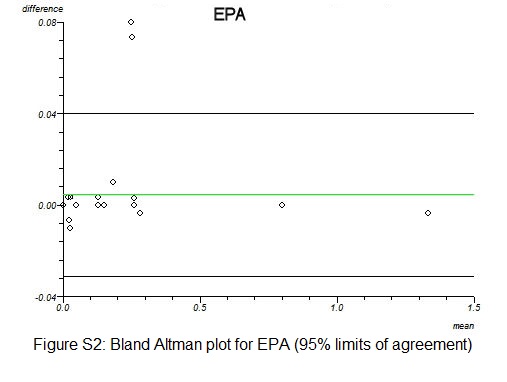

Supplement: Supplement 2 [file TVST-16-0395-s02.jpg]

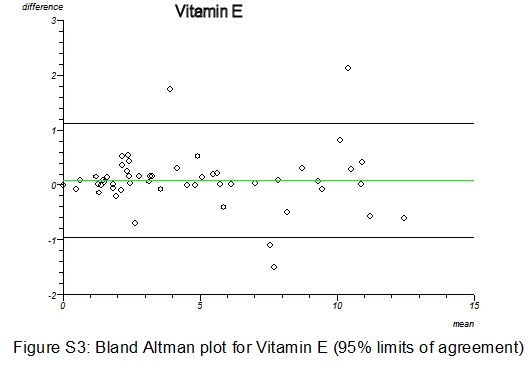

Supplement: Supplement 3 [file TVST-16-0395-s03.jpg]

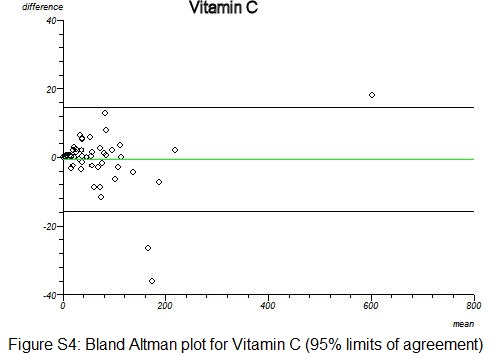

Supplement: Supplement 4 [file TVST-16-0395-s04.jpg]

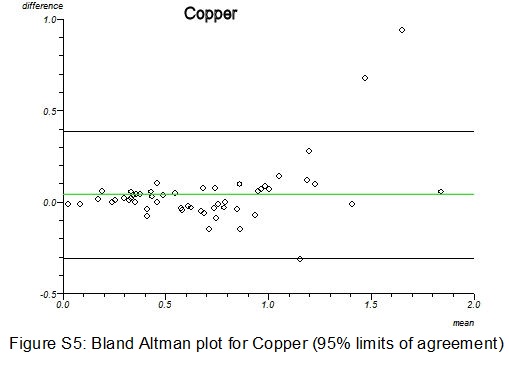

Supplement: Supplement 5 [file TVST-16-0395-s05.jpg]

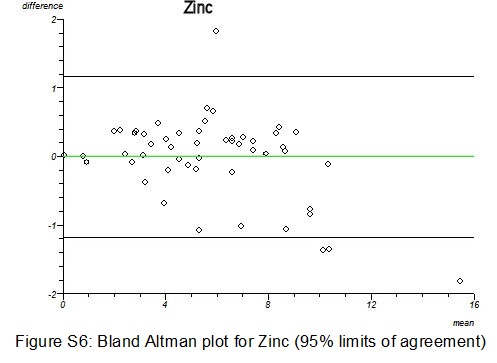

Supplement: Supplement 6 [file TVST-16-0395-s06.jpg]

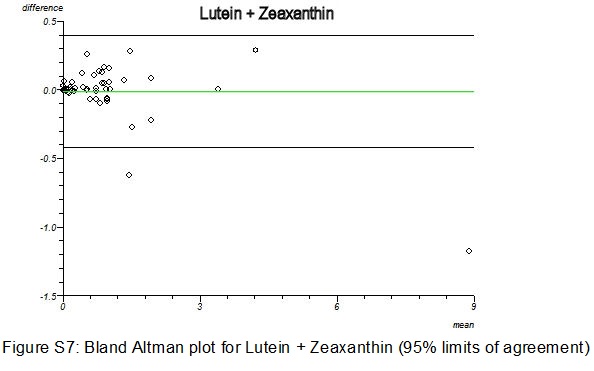

Supplement: Supplement 7 [file TVST-16-0395-s07.jpg]
